# Loss of *Rictor* in Monocyte/Macrophages Suppresses Their Proliferation and Viability Reducing Atherosclerosis in LDLR Null Mice

**DOI:** 10.3389/fimmu.2018.00215

**Published:** 2018-02-13

**Authors:** Vladimir R. Babaev, Jiansheng Huang, Lei Ding, Youmin Zhang, James M. May, MacRae F. Linton

**Affiliations:** ^1^Atherosclerosis Research Unit, Division of Cardiovascular Medicine, Department of Medicine, Vanderbilt University School of Medicine, Nashville, TN, United States; ^2^Department of Pharmacology, Vanderbilt University School of Medicine, Nashville, TN, United States

**Keywords:** atherosclerosis, monocytes, macrophages, proliferation, apoptosis, Akt signaling, mammalian target of rapamycin complex 2

## Abstract

**Background:**

Rictor is an essential component of mammalian target of rapamycin (mTOR) complex 2 (mTORC2), a conserved serine/threonine kinase that may play a role in cell proliferation, survival and innate or adaptive immune responses. Genetic loss of *Rictor* inactivates mTORC2, which directly activates Akt S^473^ phosphorylation and promotes pro-survival cell signaling and proliferation.

**Methods and results:**

To study the role of mTORC2 signaling in monocytes and macrophages, we generated mice with myeloid lineage-specific *Rictor* deletion (M*Rictor*^−/−^). These M*Rictor*^−/−^ mice exhibited dramatic reductions of white blood cells, B-cells, T-cells, and monocytes but had similar levels of neutrophils compared to control *Rictor* flox-flox (*Rictor*^fl/fl^) mice. M*Rictor*^−/−^ bone marrow monocytes and peritoneal macrophages expressed reduced levels of mTORC2 signaling and decreased Akt S^473^ phosphorylation, and they displayed significantly less proliferation than control *Rictor*^fl/fl^ cells. In addition, blood monocytes and peritoneal macrophages isolated from M*Rictor*^−/−^ mice were significantly more sensitive to pro-apoptotic stimuli. In response to LPS, M*Rictor*^−/−^ macrophages exhibited the M1 phenotype with higher levels of pro-inflammatory gene expression and lower levels of *Il10* gene expression than control *Rictor*^fl/fl^ cells. Further suppression of LPS-stimulated Akt signaling with a low dose of an Akt inhibitor, increased inflammatory gene expression in macrophages, but genetic inactivation of *Raptor* reversed this rise, indicating that mTORC1 mediates this increase of inflammatory gene expression. Next, to elucidate whether mTORC2 has an impact on atherosclerosis *in vivo*, female and male *Ldlr* null mice were reconstituted with bone marrow from M*Rictor*^−/−^ or *Rictor*^fl/fl^ mice. After 10 weeks of the Western diet, there were no differences between the recipients of the same gender in body weight, blood glucose or plasma lipid levels. However, both female and male M*Rictor*^−/−^ → *Ldlr*^−/−^ mice developed smaller atherosclerotic lesions in the distal and proximal aorta. These lesions contained less macrophage area and more apoptosis than lesions of control *Rictor*^fl/fl^ → *Ldlr*^−/−^ mice. Thus, loss of *Rictor* and, consequently, mTORC2 significantly compromised monocyte/macrophage survival, and this markedly diminished early atherosclerosis in *Ldlr*^−/−^ mice.

**Conclusion:**

Our results demonstrate that mTORC2 is a key signaling regulator of macrophage survival and its depletion suppresses early atherosclerosis.

## Introduction

Atherosclerosis, the underlying cause of myocardial infarction and stroke, is an inflammatory disease process, and both the innate and adaptive immune responses play important roles in its pathogenesis (1). Monocytes and macrophages play key roles in the pathogenesis of atherosclerosis. Furthermore, alterations in macrophage phenotypes and survival play crucial roles in the development of atherosclerosis. Current studies of immunotherapy have focused on immune checkpoint therapy targeting regulatory pathways in T-cells (2). However, there are also multiple ways to modulate the immune response by changing the activation of myeloid cells, which belong to the innate immune system, performing essential roles in tissue homeostasis by initiating, sustaining, or inhibiting adaptive immunity (3, 4). Therefore, identification of mechanisms that control macrophage metabolism and survival should provide insights into the regulation of immune responses and identify novel approaches to treat disease (5).

Recent studies suggest that interplay between protein kinase B (or Akt) and mammalian target of rapamycin (mTOR) pathways is crucial for myeloid cell activation (4). Akt is a serine/threonine-specific kinase that regulates multiple cellular processes, including cell viability, proliferation, transcription, and migration (6, 7). mTOR is an evolutionarily conserved serine/threonine kinase that is constitutively expressed and plays a central role in integrating environmental cues in the form of growth factors, amino acids, and energy (8). mTOR also controls and shapes the effector responses of innate immune cells. Therefore, knowledge of how Akt/mTOR signaling coordinates the responses of myeloid cells may provide important insights into immunity in health and disease (3, 4).

There are two distinct types of mTOR complexes, termed mTORC1 and mTOR complex 2 (mTORC2), the first contains regulatory associated protein of mTOR (Raptor) and the second involves rapamycin-insensitive companion of mTOR (Rictor) (9). Inhibition of mTORC1 with a class of macrolide immunosuppressive drugs, called rapalogs, reduces atherosclerosis in a variety of animal models, and rapalog eluting stents are widely used to treat atherosclerotic coronary artery disease in humans (10). By contrast, relatively little is known about the role of mTOR2 in atherosclerosis.

Genetic loss of *Rictor* inactivates mTORC2, and, since total *Rictor* deficiency in mice causes embryonic lethality (11, 12), generation of mice with tissue-specific knockouts of *Rictor* has been instrumental in delineating the specific roles of mTORC2 in different cell types. For example, liver-specific disruption of *Rictor* induces glucose intolerance, hepatic insulin resistance, and decreased hepatic lipogenesis (13, 14). Remarkably, the liver-specific knockout or an inducible total body loss of *Rictor* in adult mice specifically reduces male lifespan (15). In contrast, *Rictor* knockout in muscle tissue contributes to glucose homeostasis by positively regulating insulin-stimulated glucose uptake and negatively regulating basal glycogen synthase activity (16). mTORC2 regulates cardiomyocyte growth and survival, and loss of *Rictor* in mouse heart results in progressive cardiac dysfunction (17). Adipose-specific knockout of *Rictor* in mice increases whole-body size (18) but leads to hepatic steatosis and insulin resistance (19). Loss of *Rictor* in pancreatic beta cells reduces beta cell mass and insulin secretion resulting in hyperglycemia and glucose intolerance (20). These data indicate that mTORC2 may play varied roles depending on the cell type and conditions.

Notably, mTORC2 initiates the phosphorylation of Akt S^473^ (21), which accelerates Akt signaling promoting cell survival and proliferation. Despite the predominant role of mTORC2 as a regulator of pro-survival Akt signaling, the impact of mTORC2 on cell viability remains unclear (22). For example, several studies showed no indication that loss of *Rictor* and consequently mTORC2 increased apoptosis, at least under otherwise normal physiological conditions (16, 18, 20, 23). Moreover, a recent report indicates that *Rictor*-deficient keratinocytes display increased lifespan, protection from senescence, and enhanced tolerance of cellular stressors such as growth factor deprivation (24). These findings appear to contradict several other reports indicating that *Rictor* deficiency increases apoptosis in pulmonary arterial vascular smooth muscle cells (25), endothelial cells (26) and skin tumor cells (27). Rictor plays an important role in regulating cellular survival after ischemic cardiac damage (28) and in B-cells acting via a c-Myc-dependent pathway (29). In addition, we recently demonstrated that loss of IκB kinase alpha (IKKα) in macrophages suppresses Akt S^473^ phosphorylation and this compromises cell survival and decreases early atherosclerosis (30). This suppression of p-Akt S^473^ was mediated *via* mTORC2 suggesting the hypothesis that mTORC2 initiates important pro-survival signaling in monocytes and macrophages that impacts the pathogenesis of atherosclerosis. However, IKKα is located upstream of the NF-κB signal transduction cascade and loss of *IKK*α-enhanced macrophage activation (31), limiting the ability to draw conclusions regarding the role of mTORC2 in macrophage function and atherogenesis.

Therefore, we used myeloid lineage-specific *Rictor* deletion in mice to examine directly whether loss of mTORC2 in myeloid cells impacts monocyte and macrophage viability and proliferation and the development of atherosclerosis. We found that mTORC2 signaling is crucial for monocyte and macrophage proliferation and survival, and loss of mTORC2 signaling in myeloid cells results in decreased early atherosclerosis.

## Materials and Methods

### Animal Procedures, Mice

*Rictor*^flox/flox^ (32), *LysM-Cre* (33), *Raptor*^flox/flox^ (34), and the recipient LDLR^−/−^ mice were all on the C57BL/6 background, and they were purchased from the Jackson Laboratories together with C57BL/6 mice. Mice with myeloid lineage-specific *Raptor* deletion (M*Raptor*^−/−^) were generated by crossing *Raptor* floxed mice (*Raptor*^flox/flox^) with LysM-Cre mice. Mice were maintained in microisolator cages on a rodent chow diet containing 4.5% fat (PMI 5010, St. Louis, MO, USA) or a Western-type diet containing 21% milk fat and 0.15% cholesterol (Teklad, Madison, WI, USA).

### Ethics Statement

All animal experiments were approved by the Institutional Animal Care and Use Committee at Vanderbilt University Medical Center. Vanderbilt University Medical Center Animal Care and Use Program is registered with the USDA (USDA Registration #63-R-0129) and operates under a PHS Animal Welfare Assurance Statement (PHS Assurance #A3227-01).

### Bone Marrow Transplantation, Blood Monocyte, and Peritoneal Macrophage Isolation

Bone marrow cells were isolated from mouse tibias and fibulas. Recipient *Ldlr*^−/−^ or C57BL6 mice were lethally irradiated (9 Gy) and transplanted with (5 × 10^6^) bone marrow cells as described (35). Blood was collected by retro-orbital bleeding under anesthesia, and peripheral blood mononuclear cells were isolated by centrifugation in Histopaque-1077 (Sigma). Cells that attached to the plastic dish within 2–3 h of incubation were cultured in DMEM media containing 10% FBS and macrophage colony-stimulating factor (M-CSF; 10 ng/ml). Thioglycollate-elicited peritoneal macrophages were isolated and treated with insulin from bovine pancreas, or fatty acid free bovine serum albumin (BSA) (both from Sigma), or with palmitic acid complexed to BSA (PA-BSA), human oxidized LDL, or an Akt inhibitor IV (EMD Millipore).

### Western Blotting

Cells were lysed in a lysis buffer (Cell Signaling Technology, Danvers, MA, USA) containing protease and phosphatase inhibitors. Proteins were measured with the DC Protein assay reagents (Bio-Rad Laboratories) and resolved by NuPAGE Bis-Tris electrophoresis and transferred onto nitrocellulose membranes (Amersham Bioscience). Blots were probed with rabbit antibodies to Akt, p-Akt (both S^473^ and T^308^), Rictor, p-mTOR S^2448^, p-70S6K T^389^, p-4E-BP1 T^37/46^ (all from Cell signaling Technology), p-SGK S^422^R and p-PKCα S^657^-R, p-NDRG1 T^346^ and β-actin antibodies (Santa Cruz Biotechnology), and goat anti-rabbit horseradish peroxidase-conjugated secondary antibodies (Sigma). Proteins were visualized with ECL western blotting detection reagents (GE Healthcare) and quantified by densitometry using ImageJ software (NIH).

### Flow Cytometry

Blood cells were stained with a cocktail of antibodies against lineage markers, including CD3 (clone 17A2), CD45RB220 (clone RA3-6B2), Ly-6G (clone 1A8), CD11b (clone M1/70), and Ly-6C (clone AL-21) (all from BioLegend). Blood or bone marrow cells were treated with lysing buffer (BD Pharm, cat#555899), washed and analyzed using FACS DiVa v6.1 software (BD Biosciences) in the Research Flow Cytometry Core Laboratory, Veterans Administration Medical Center. Akt phosphorylation levels were evaluated in bone marrow cells using a methanol-based permeabilization protocol (36).

### BrdU-Labeled Cell Proliferation Assay

For the *in vivo* experiments, BrdU labeling reagent (Invitrogen, catalog#000103) was injected intraperitoneal [10 ml/kg body weight (BW)], and, 24 h later, cells were isolated, fixed, permeabilized, treated with DNAse and then analyzed by flow cytometry using a FITC BrdU flow kit (cat#51-2354AK). For *in vitro* studies, cells were treated with or without human recombinant platelet-derived growth factor (PDGF-BB, eBisoscience; 20 ng/m) together with BrdU, diluted 1:100, for 24 h. Then cells were fixed with 2% paraformaldehyde for 20 min, permeabilized with 0.1% Triton X-100, DNA was denatured by incubation with 2 N HCl for 60 min at 37°C, followed by five rinses in 0.1 Borate buffer, pH8.5 and PBS. Cells were blocked for 30 min by using 3% BSA in PBS, probed with a fluorochrome-conjugated anti-BrdU antibody and analyzed under a microscope (Olympus AX70, camera DP72).

### Apoptosis Assessment

Apoptotic cells were analyzed by flow cytometry and in cultured cells seeded in Laboratory-Tek chambers (Nalge Nunc International) using an Alexa Fluor488 Annexin V/Dead Cell Apoptosis kit (Life Technologies, catalog number V13241). To detect apoptosis in the vascular wall, 5-μm cryostat sections of the proximal aorta were fixed in 2% paraformaldehyde in PBS, treated with 3% citric acid and stained using an *in situ* cell death detection kit (Roche Applied Science). TUNEL-positive (TUNEL+) cells were counted in four different sections of each aorta as described (37). The numbers of TUNEL+ cells were counted as a percentage of the total number of cells in at least four separate fields (containing ≈1,000 cells) from duplicate chambers.

### RNA Isolation and Real-Time PCR

Total RNA was isolated and relative quantitation of the target mRNA was analyzed using gene expression assays (Applied Biosystems, Foster City, CA, UK) as described (37).

### Analysis of Serum Lipids and Aortic Lesions

Serum total cholesterol and triglyceride (TG) levels were determined after overnight fasting by the enzyme-based technique. Aortas were flushed through the left ventricle and the entire aorta was dissected *en face* and stained with Sudan IV as described (37). Cryostat sections of aortic sinus were stained with Oil-red-O, images of the proximal and distal aortas were analyzed using an Imaging system KS 300 (Kontron Electronik GmbH.).

### Statistical Analysis

All analyses were performed using GraphPad Prizm v 7 (Graph-Pad Software, Inc., USA) or a SPSS Statistics Premium v22 (IBM, USA) software. Data were assessed for normality of distribution. Statistical analyses of data were performed using Student’s *t*-test, a Mann–Whitney *U*-test or 1- to 2-way ANOVAs. Data are provided as means ± SEM. A difference was considered to be statistically significant at a *P*-value less than 0.05 by Mann–Whitney Rank Sum Test.

## Results

### Loss of Rictor Reduces Akt and mTORC2 Signaling in Blood Monocytes and Macrophages

The stability and integrity of mTORC2 depends on the presence of Rictor, which is an essential component of the complex. Deficiency in Rictor disrupts mTORC2 assembly and this significantly diminishes cell AktS^473^ phosphorylation (11). However, total Rictor deficiency in mice causes embryonic lethality (11, 12). Therefore, tissue-specific Rictor knockout mice represent an approach for investigating the role of mTORC2 in cell-specific functions (38).

**Figure 1:**
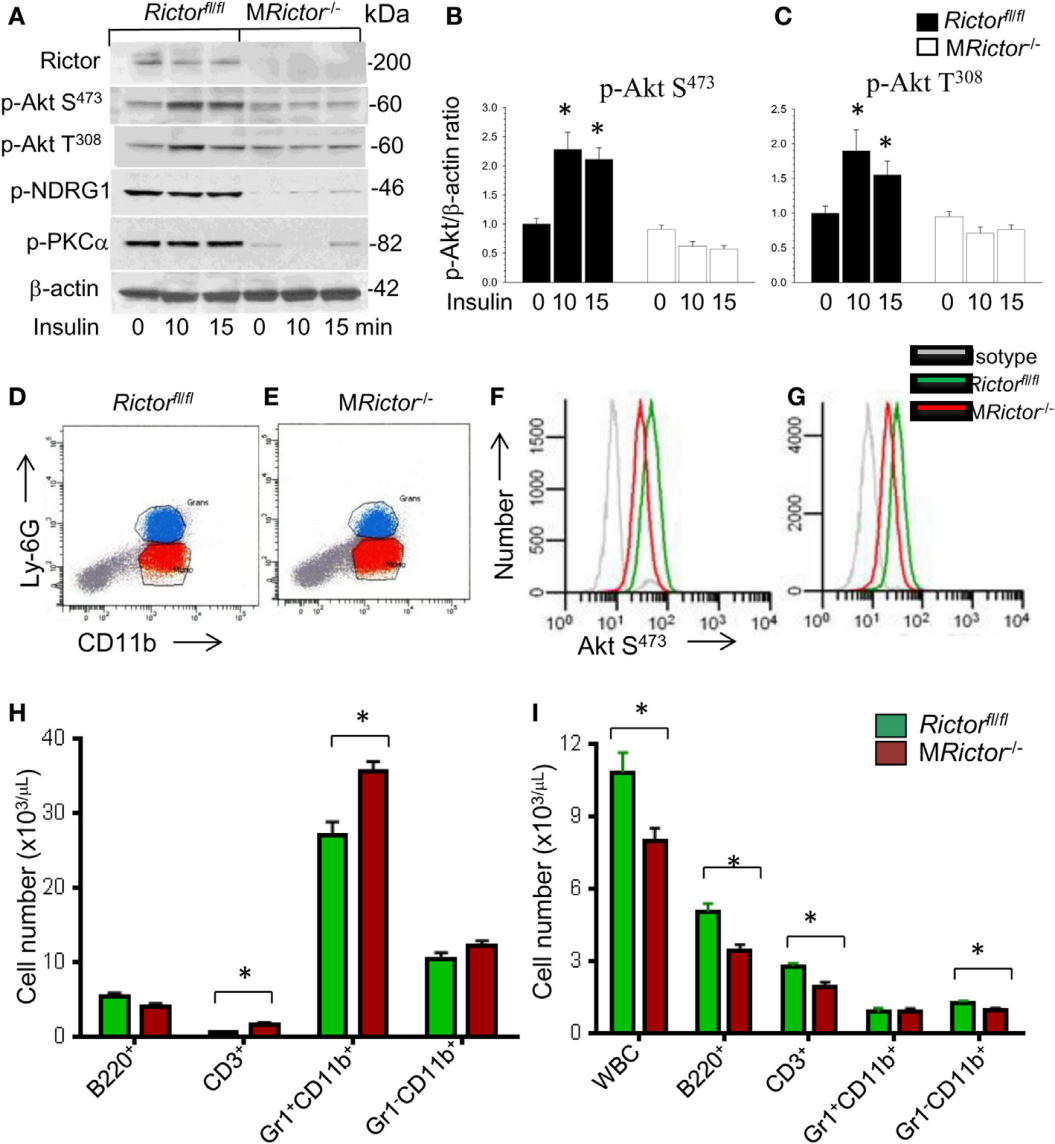
Loss of *Rictor* significantly suppresses Akt and mammalian target of rapamycin complex 2 (mTORC2) signaling in peritoneal macrophages, reduces p-Akt S^473^ in monocytes and neutrophils *in vivo*, and decreases blood leukocyte numbers. **(A–C)** Peritoneal macrophages were isolated from *Rictor*^fl/fl^ and M*Rictor*^−/−^ mice (*n* = 3/group), incubated overnight in serum-free media then untreated or treated with insulin (100 nM) for 10 and 15 min. Proteins were extracted, resolved (60 μg/well) and analyzed by western blot using the antibodies as indicated. Note the reduction of p-Akt S^473^, p-Akt^308^ and mTORC2 downstream signaling, p-NDRG1 and p-PKCα in M*Rictor*^−/−^ macrophages compared to control *Rictor*^fl/fl^ cells. Graphs represent data (mean ± SEM; *p < 0.05 compared to control untreated *Rictor*^fl/fl^ cells). **(D–G)** Representative plots of gaiting strategy to analyze Akt S^473^ phosphorylation levels in bone marrow cells of *Rictor*^fl/fl^ and M*Rictor*^−/−^ mice *in vivo* **(D,E)**. Note M*Rictor*^−/−^ bone marrow neutrophils **(F)** and monocytes **(G)** expressed significantly less p-Akt S^473^ (red) than control *Rictor*^fl/fl^ cells (green) but were higher than the isotype control antibodies (gray). **(H,I)** Multicolor flow cytometry analysis of bone marrow **(H)** and blood cells **(I)** isolated from *Rictor*^fl/fl^ (green) and *MRictor*^−/−^ (red) mice. Note the increase of T-cells and neutrophils in bone marrow and decrease of white blood cells, B- and T-cells and monocytes but not granulocytes in blood (mean ± SEM; *p < 0.05 compared to control sample). These experiments were repeated three times.

To elucidate the role of mTORC2 signaling in monocytes and macrophages, we generated mice with myeloid lineage-specific *Rictor* deletion by crossing *Rictor* floxed mice (*Rictor*^fl/fl^) with lysozyme M Cre (*LysM-Cre*)–recombinase mice. These myeloid-specific *Rictor* knockout *(*M*Rictor*^−/−^) mice were born at the expected Mendelian ratio and were viable and fertile with no differences in BW or plasma lipid levels compared to control *Rictor*^fl/fl^ mice. Analysis of peritoneal macrophages isolated from M*Rictor*^−/−^mice demonstrated that these cells had significantly less Rictor protein than control *Rictor*^fl/fl^ cells (Figure 1A). In response to insulin treatment, Akt S^473^ and Akt T^308^ phosphorylation were significantly suppressed in M*Rictor*^−/−^ macrophages compared to *Rictor*^fl/fl^ cells (Figures 1A–C). In addition, M*Rictor*^−/−^ macrophages had diminished levels of proteins known to be downstream targets of mTORC2. The levels of protein kinase C alpha and the serum and glucocorticoid-induced kinase 1 substrate, N-myc downstream regulated gene 1 (NDRG1), were only slightly detectable in M*Rictor*^−/−^ macrophages compared to *Rictor*^fl/fl^ cells (Figure 1A). Together, these results are consistent with our expectation that loss of *Rictor* prevents mTORC2 formation and this significantly suppresses p-Akt S^473^ and Akt signaling.

In order to evaluate whether *Rictor* deficiency suppresses Akt signaling *in vivo*, we analyzed Akt S^473^ phosphorylation in mouse bone marrow cells by flow cytometry using antibodies to CD11b and Ly-6G to separate monocytes and neutrophils (Figures 1D,E). As we anticipated, M*Rictor*^−/−^ granulocytes and monocytes expressed decreased levels of Akt Ser^473^ phosphorylation compared to control *Rictor*^fl/fl^ cells (Figures 1F,G). These results confirm that *Rictor* deletion in myeloid cells significantly suppresses Akt signaling *in vivo*.

Next, to examine the impact of myeloid *Rictor* deficiency on bone marrow and blood cells, we used multicolor flow cytometry analysis. Interestingly, we isolated similar total numbers of bone marrow cells from *Rictor*^fl/fl^ and M*Rictor*^−/−^ mice (*n* = 18/group; 47.1 ± 2.1 vs. 45.8 ± 2.8 × 10^6^ cells, respectively; *p* = 0.71). However, bone marrow cells from M*Rictor*^−/−^ mice showed increased numbers of T-cells and neutrophils, but no changes in B-cells or monocytes compared to bone marrow of *Rictor*^fl/fl^ mice (Figure 1H). By contrast, blood cells from M*Rictor*^−/−^ mice had significantly lower numbers of total white blood cell counts, decreased B-cells, T-cells, and monocytes, but not neutrophils, than *Rictor*^fl/fl^ mice (Figure 1I). These data indicate that mTORC2 is an important determinant of blood cell numbers including monocytes. These findings also suggest the loss of mTORC2 may influence hematopoietic cell proliferation and viability.

### Rictor Is Necessary for Proliferation of Myeloid Cells

**Figure 2:**
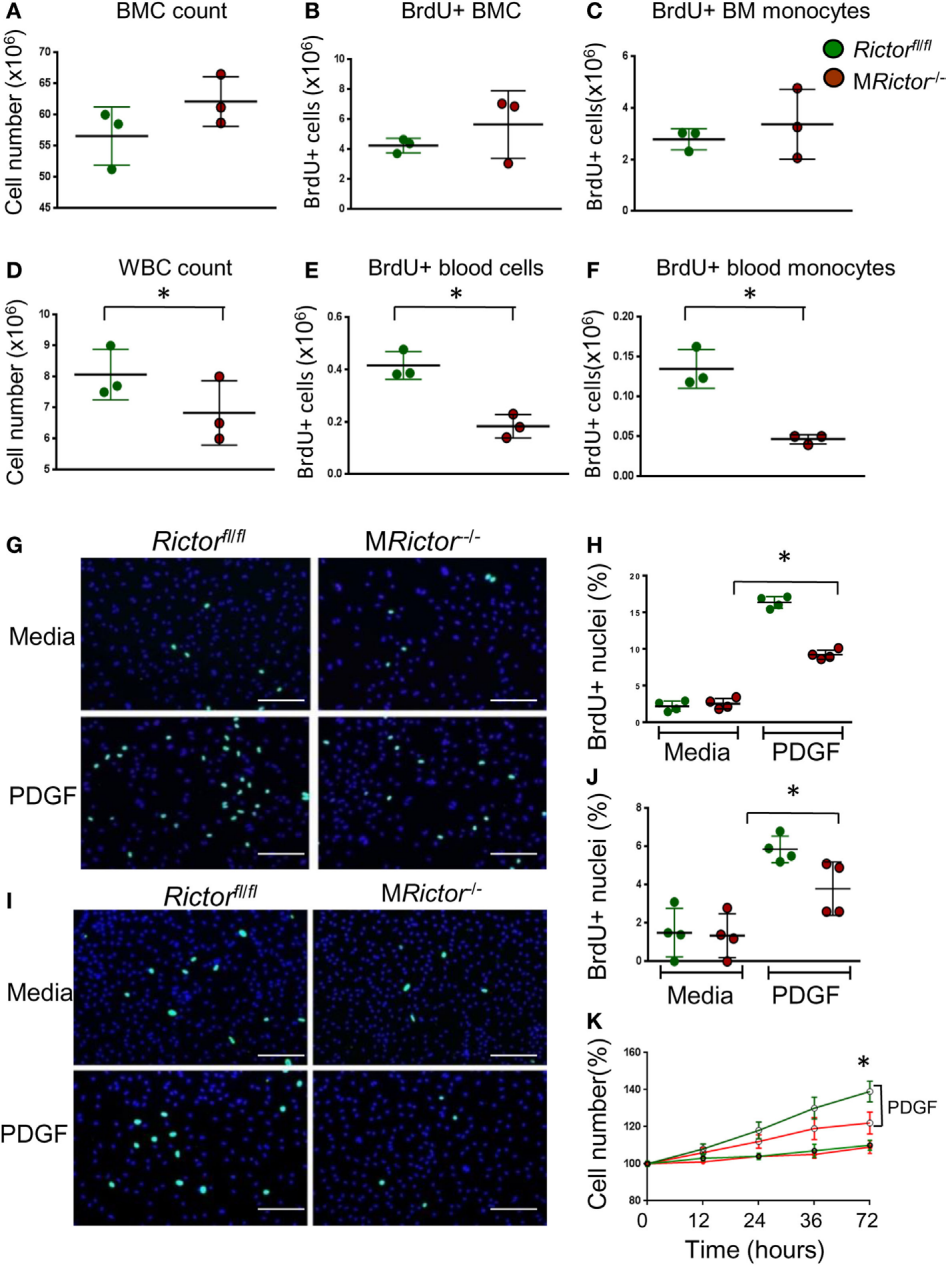
Loss of *Rictor* suppresses proliferation of blood monocytes and macrophages. **(A–F)** BrdU (10 ml/kg) was I/P injected into *Rictor*^fl/fl^ and M*Rictor*^−/−^ mice, 24 h later bone marrow and blood cells were isolated and analyzed by flow cytometry. Note there were no differences in bone marrow cell count, total BrdU+ cells and BrdU+ monocytes **(A–C)**; however, white blood cell count, total BrdU+ blood cells and BrdU+ monocytes were reduced in M*Rictor*^−/−^ compared to *Rictor*^fl/fl^ mice. Graphs represent data (mean ± SEM) of the experiment with three mice per group (*p < 0.05 compared to control untreated group). **(G–J)** Blood monocytes **(G,H)** and peritoneal macrophages **(I,J)** were isolated from *Rictor*^fl/fl^ and M*Rictor*^−/−^ mice, and two days incubation in DMEM media containing 10% FBS (and M-CSF for monocytes) treated with or without PDGF (20 ng/ml) and BrdU overnight. The incorporation of BrdU was analyzed under a fluorescent microscopy. Note PDGF treatment significantly increased proliferation but less prominent in M*Rictor*^−/−^ than in *Rictor*^fl/fl^ cells. Graphs represent data (mean ± SEM) obtained from different mice (*n* = 4/group; *p < 0.05 *t*-test analysis compared to *Rictor*^fl/fl^ cells); Scale bar is 50 mm. **(K)** WT and M*Rictor*^−/−^ peritoneal macrophages were seeded in triplicate on a 48-well plate and then treated with DMEM media alone or together with PDGF (20 ng/ml) for the indicated times. The cells were counted using an EVOS FL Auto imaging System (Life Technology). Note, top two lines of the graph represent cells treated with PDGF, as indicated.

Since one of the distinct characteristic of macrophages is self-renewal via proliferation (39), we examined whether elimination of mTORC2 disturbs the proliferation of monocytes and macrophages. The BrdU incorporation rate was analyzed in bone marrow and blood cells of *Rictor*^fl/fl^ and M*Rictor*^−/−^ mice. There were no differences between these groups of mice in total bone marrow cells, total number of BrdU-positive (BrdU+) marrow cells and BrdU+ monocytes (Figures 2A–C). In contrast, blood cells of M*Rictor*^−/−^ mice had lower levels of white blood cell counts, total number of BrdU+ cells and BrdU+monocytes compared to control *Rictor*^fl/fl^ mice (Figures 2D–F). Thus, loss of *Rictor* diminishes numbers of proliferative monocytes in the blood but not in the bone marrow.

To test whether M*Rictor*^−/−^ blood monocytes respond appropriately to a proliferative stimulus, we isolated blood monocytes from *Rictor*^fl/fl^ and M*Rictor*^−/−^ mice and 2 days later treated them with PDGF. The treatment increased numbers of BrdU+ cells in both groups although to a significantly greater degree in *Rictor*^fl/fl^ monocytes than in M*Rictor*^−/−^ cells (Figures 2G,H). Similarly, peritoneal macrophages isolated from M*Rictor*^−/−^ mice and treated with PDGF also exhibited diminished numbers of BrdU+ cells compared to M*Rictor*^fl/fl^ cells (Figures 2I,J). Correspondingly, a simple cell count after treatment with PDGF revealed a greater increase of *Rictor*^fl/fl^ macrophages compared to M*Rictor*^−/−^ cells (Figure 2K). Collectively, these results indicate that mTORC2 is required for proliferation of monocytes and macrophages.

### M*Rictor*^−/−^ Macrophages Are Less Viable and Express High Levels of Inflammatory Genes in Response to LPS

**Figure 3:**
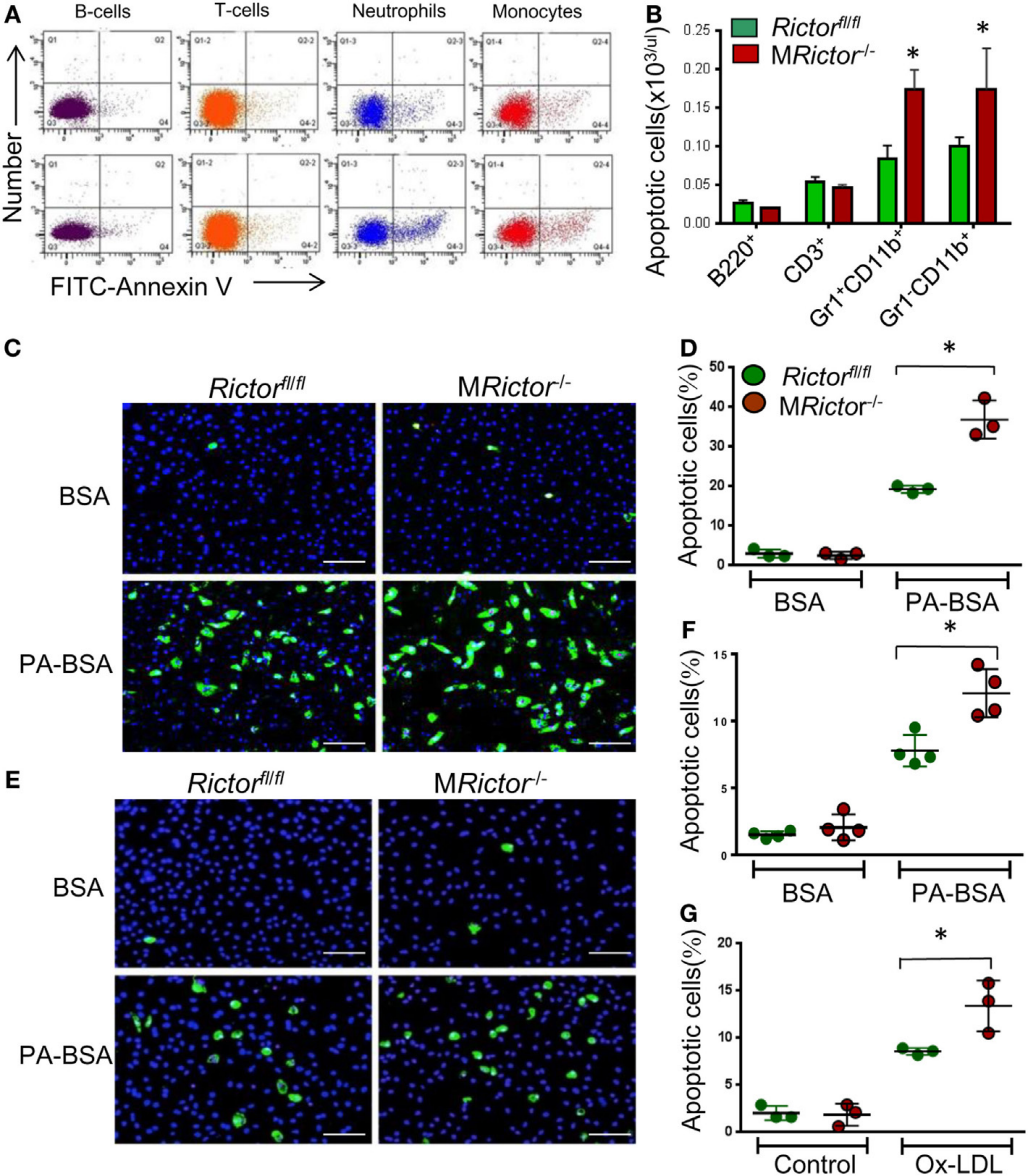
M*Rictor*^−/−^ macrophages are less resistant to different pro-apoptotic stimuli than *Rictor*^fl/fl^ cells. **(A,B)** Representative flow cytometry plots of apoptotic blood cells isolated from *Rictor*^fl/fl^ (top panel) and M*Rictor*^−/−^ mice (bottom panel) and stained with the Alexa Fluor 488 Annexin V. Note increase of apoptosis in blood monocytes and neutrophils of M*Rictor*^−/−^ mice compared to *Rictor*^fl/fl^ mice. Graphs represent data (mean ± SEM) of the experiment (*n* = 3/group, *p < 0.05 *t*-test analysis compared to *Rictor*^fl/fl^ mice). **(C,D)** Blood monocytes were isolated from *Rictor*^fl/fll^ and M*Rictor*^−/−^ mice (*n* = 3/group), incubated in DMEM media containing 10% FBS, M-CSF for 2 days, and then treated overnight with 0.3 M palmitic acid complexed to bovine serum albumin (PA-BSA) in the presence of 1% FBS and M-CSF. The Alexa Flour 488 Nalge Nunc International/dead cell apoptosis kit was used to detect apoptotic cells. Note PA-BSA treatment significantly increased apoptosis in both group of cells, more prominently in M*Rictor*^−/−^ than *Rictor*^fl/fl^ cells; scale bars, 50 μm. Graphs represent data (mean ± SEM; *p < 0.05 by Mann–Whitney rank sum test). **(E,G)** Peritoneal macrophages were isolated from *Rictor*^fl/fl^ and M*Rictor*^−/−^ mice (*n* = 4/group), and 2 days later treated with 0.5 M PA-BSA overnight **(E,F)** or human oxidized LDL (100 μg/ml) for 24 h **(G)**. Annexin V/dead cell apoptosis kit was used to detect apoptotic cells; Note PA-BSA increases apoptosis more obviously in M*Rictor*^−/−^ than *Rictor*^fl/fl^ macrophages; scale bars, 50 μm. Graphs represent data (mean ± SEM) of four mice/group; *p < 0.05 by Mann–Whitney rank sum test.

Since loss of *Rictor* disrupts assembly of mTORC2, thus significantly diminishing Akt signaling, which is the major pro-survival pathway that suppresses apoptosis (6), we hypothesized that *Rictor* deficiency may compromise monocyte and macrophage survival. To test this hypothesis, we analyzed blood cells isolated from *Rictor*^fl/fl^ *and* M*Rictor*^−/−^ mice by flow cytometry using the Alexa Fluor488 Annexin V antibody. The analysis of the representative flow cytometry plots revealed that M*Rictor*^−/−^ mice had an increase of apoptosis in blood neutrophils and monocytes but not in B- or T-cells compared to *Rictor*^fl/fl^ mice (Figure 3A). Indeed, M*Rictor*^−/−^ mice had increased apoptotic neutrophils and monocytes but not B- and T-cells compared to *Rictor*^fl/fl^ mice (Figure 3B). Thus, loss of mTORC2 significantly increases apoptosis in blood neutrophils and monocytes.

To further investigate this phenomenon, we isolated blood monocytes and treated them overnight with BSA or BSA complexed with palmitic acid (PA), a lipotoxic factor that induces ER stress and triggers apoptosis (40). Analysis of apoptosis by the Annexin V labeling kit demonstrated that PA treatment dramatically increased apoptosis in both groups of cells although more notably in M*Rictor*^−/−^ monocytes (91%) than *Rictor*^fl/fl^ cells (Figures 3C,D). Thus, M*Rictor*^−/−^ monocytes exhibit compromised resistance to ER stress-induced apoptosis. Similar results were obtained when peritoneal macrophages were treated with PA or human oxidized LDL, which may be more relevant to atherosclerosis, M*Rictor*^−/−^ macrophages had significantly higher levels of apoptosis than *Rictor*^fl/fl^ cells (Figures 3E–G). Together these data indicate that loss of *Rictor* and consequently mTORC2 significantly compromises the ability of monocytes and macrophages to survive under conditions of ER stress.

**Figure 4:**
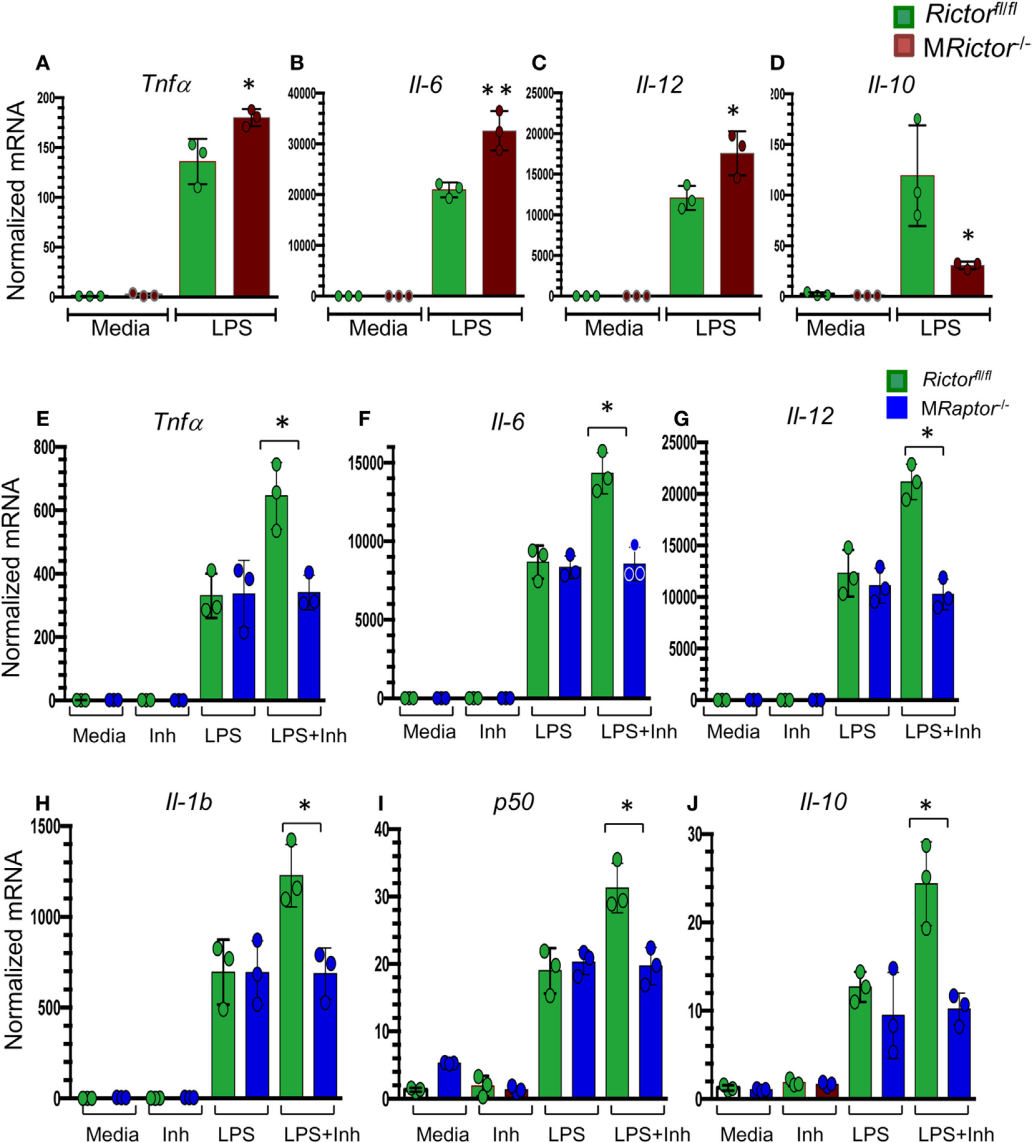
M*Rictor*^−/−^ macrophages have increased levels of inflammatory gene expression in response to lipopolysaccharide (LPS) and a low dose of Akt inhibitor increases the LPS-induced expression of inflammatory genes in *Rictor*^fl/fl^ but not in M*Raptor*^−/−^ macrophages. **(A–D)** Peritoneal macrophages were incubated with media alone (control) or together with LPS (20 ng/ml) for 5 h and the gene-expression levels were measured by real-time polymerase chain reaction. Note M*Rictor*^−/−^ macrophages have increased pro-inflammatory and decreased of interleukin-10 gene expression. Graphs represent data (mean ± SEM) obtained from the same numbers (*n* = 3 per group) of mice (*p < 0.05 and **p < 0.001 compared to *Rictor*^fl/fl^ cells treated with LPS by *t*-test). **(E–J)** Peritoneal macrophages were isolated from *Rictor*^fl/fl^ and M*Raptor*^−/−^ mice and 2 days later were treated with media (Control), with the Akt inhibitor IV (31 μM, Inh) or LPS (20 ng/ml) alone or together with the inhibitor (LPS + Inh) for 5 h at 37°C and the gene-expression levels were measured by real-time polymerase chain reaction. Note a low dose of the Akt inhibitor increases LPS-mediated inflammatory gene expression but loss of Raptor reverses the increase. Graphs represent data (mean ± SEM) obtained from the same numbers (*n* = 3 per group) of mice (*p < 0.05 and **p < 0.001 compared to WT cells treated with LPS by *t*-test test).

Recently, several reports (41–43) have shown that mTORC2 signaling is crucial in promoting the M2 phenotype. To verify these findings, M*Rictor*^−/−^ *Rictor*^fl/fl^ peritoneal macrophages were treated with lipopolysaccharides (LPS) and analyzed by real-time PCR. Accordingly, M*Rictor*^−/−^ macrophages expressed significantly higher levels of inflammatory genes including *TNF*α, *Il6*, and *Il12a* than *Rictor*^fl/fl^ cells (Figures 4A–C). At the same time, the level of *Il10 g*ene expression was significantly lower in M*Rictor*^−/−^ than in *Rictor*^fl/fl^ macrophages (Figure 4D). In addition, we demonstrated that suppression of Akt signaling with a low dose of an Akt inhibitor further increased expression of these inflammatory cytokines (Figures 4E–J). Importantly, genetic loss of *Raptor*, which is an essential member of mTORC1, eliminated this increase, indicating that mTORC1 is responsible for the increase of expression of inflammatory genes (Figures 4E–J). Thus, our results demonstrate that deletion of mTORC2 inhibits M2 polarization, and the levels of Akt signaling are important in this process.

### Loss of Rictor in Hematopoietic Cells Reduces Early Atherosclerosis in Female and Male *Ldlr*^−*/*−^ Mice

**Figure 5:**
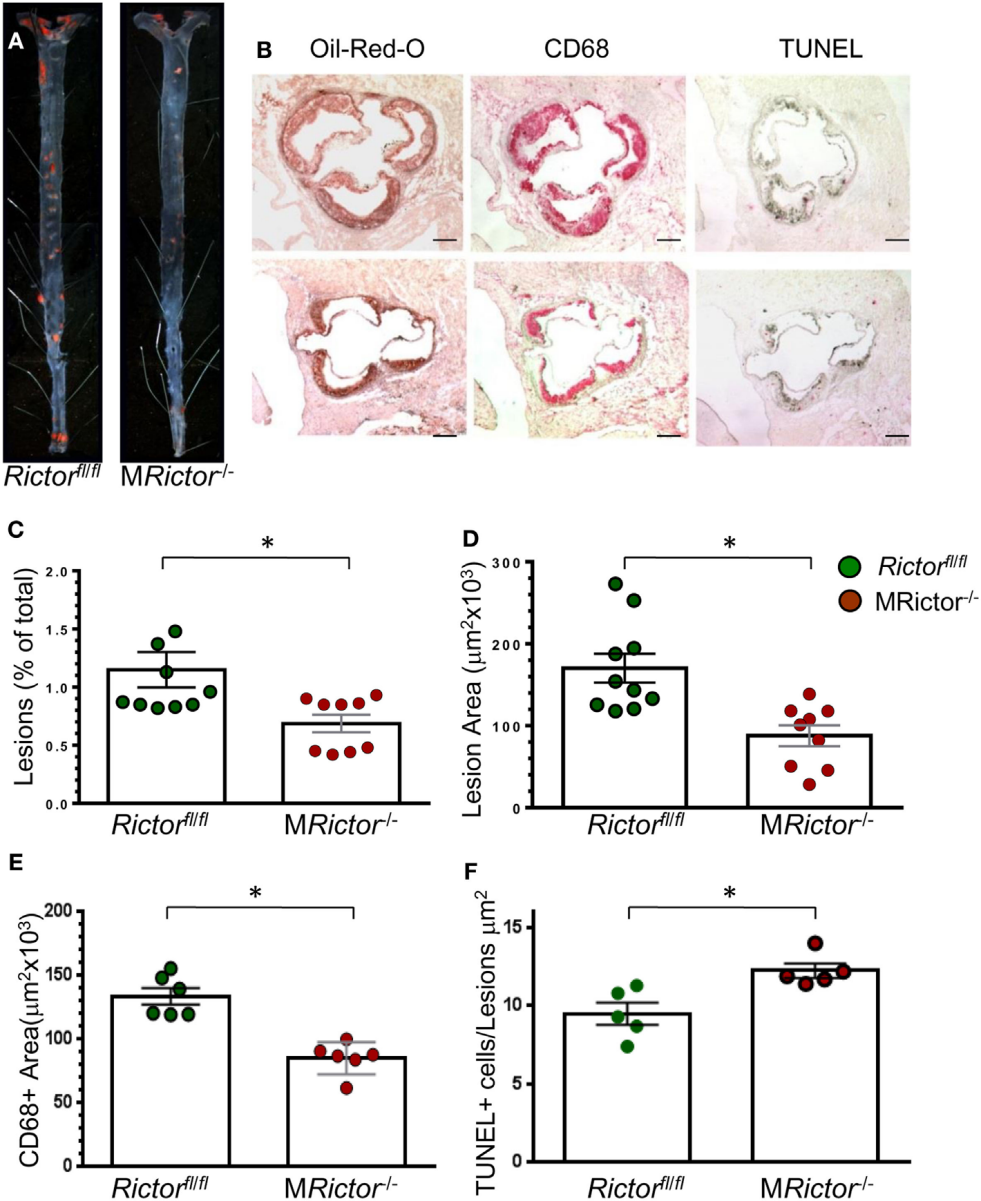
Female M*Rictor*^−/−^ → *Ldlr*^−/−^ mice had smaller atherosclerotic lesions, less macrophage area and more apoptotic cells in the lesions than control *Rictor*^fl/fl^ → *Ldlr*^−/−^ mice. **(A)** Representative images of and Sudan IV-stained en face preparation of aortas and **(B)** and serial cross sections of aortic sinus stained with Oil-Red-O/hematoxylin, CD68 and TUNEL AP from *Rictor*^fl/fl^ → *Ldlr*^−/−^ mice (B, top panel) and M*Rictor*^−/−^ → *Ldlr*^−/−^ mice (B, bottom panel) mice. Scale bars, 200 μm. **(C,D)** Quantitation of atherosclerotic lesions in aortas en face and cross sections of aortic sinus of *Rictor*^fl/fl^ → *Ldlr*^−/−^ and M*Rictor*^−/−^ → *Ldlr*^−/−^ bone marrow cells; *p < 0.05 by Mann–Whitney rank sum test. **(E,F)** Macrophage area stained with CD68 and number of TUNEL + cells in atherosclerotic lesions of mice reconstituted with WT or M*Rictor*^−/−^ bone marrow cells; *p < 0.05 by *t*-test.

To evaluate whether loss of mTORC2 signaling in macrophages has an impact on atherosclerosis *in vivo*, we generated female and male chimeric *Ldlr*^−/−^ mice with M*Rictor*^−/−^ or *Rictor*^fl/fl^ hematopoietic cells using bone marrow transplantation (35). In the first experiment, 19-week-old female *Ldlr*^−/−^ mice were lethally irradiated and transplanted with female *Rictor*^fl/fl^ (*n* = 10) or M*Rictor*^−/−^(*n* = 9) bone marrow cells. After 4 weeks, the recipient mice were fed with the Western-type diet for 10 weeks. There were no statistically significant differences in BW, serum total cholesterol or TG levels between the groups (Table 1). However, the recipients of M*Rictor*^−/−^ bone marrow cells had 32% smaller atherosclerotic lesions in the *en face* analysis compared to mice reconstituted with *Rictor*^fl/fl^ marrow cells (Figures 5A,C). Similarly, M*Rictor*^−/−^ → *Ldlr*^−/−^ mice had 28% smaller atherosclerotic lesions in the cross sections of aortic sinus compared to control mice with *Rictor*^fl/fl^ marrow (Figures 5B,D). The lesions of M*Rictor*^−/−^ → *Ldlr*^−/−^ mice had a smaller macrophage area stained with antibody to CD68 than lesions of *Rictor*^fl/fl^ → *Ldlr*^−/−^ mice (Figure 5E). Remarkably, M*Rictor*^−/−^ → *Ldlr*^−/−^ mice had significantly more TUNEL-positive cells in atherosclerotic lesions than control *Rictor*^fl/fl^ → *Ldlr*^−/−^ mice (Figure 5F). Thus, loss of mTORC2 signaling in hematopoietic cells significantly increases macrophage apoptosis in atherosclerotic lesions and this reduces the extent of atherosclerosis in *Rictor*^fl/fl^ → *Ldlr*^−/−^ mice.

**Table 1:** Body weight (BW), blood glucose (BG), total serum cholesterol (TC), and triglyceride (TG) levels in *Ldlr*^−/−^ mice reconstituted with female (A) and male (B) *Rictor*^fl/fl^ and M*Rictor*^−/−^ bone marrow cells after 10 weeks of the Western diet.

Recipient mice	BW (g)	BG (mg/dL)	TC (mg/dL)	TG (mg/dL)
**(A) Females**				
*Rictor*^fl/fl^ (*n* = 10)	22.7 ± 0.7	163.5 ± 4.4	935 ± 33	203 ± 17
M*Rictor*^−/−^ (*n* = 10)	22.2 ± 0.9	156.2 ± 5.0	949 ± 52	187 ± 18
*p*	0.91	0.21	0.87	0.90
**(B) Males**				
*Rictor*^fl/fl^ (*n* = 10)	23.4 ± 0.2	139.7 ± 4.7	750 ± 38	158 ± 28
M*Rictor*^−/−^ (*n* = 9)	22.6 ± 0.6	151.1 ± 9.9	714 ± 22	131 ± 19
*p*	0.73	0.58	0.41	0.59

*Values are (Mean ± SEM). The number of recipient mice in each group is indicated by *n*. *p* = the differences are not statistically significant between the groups*.

**Figure 6:**
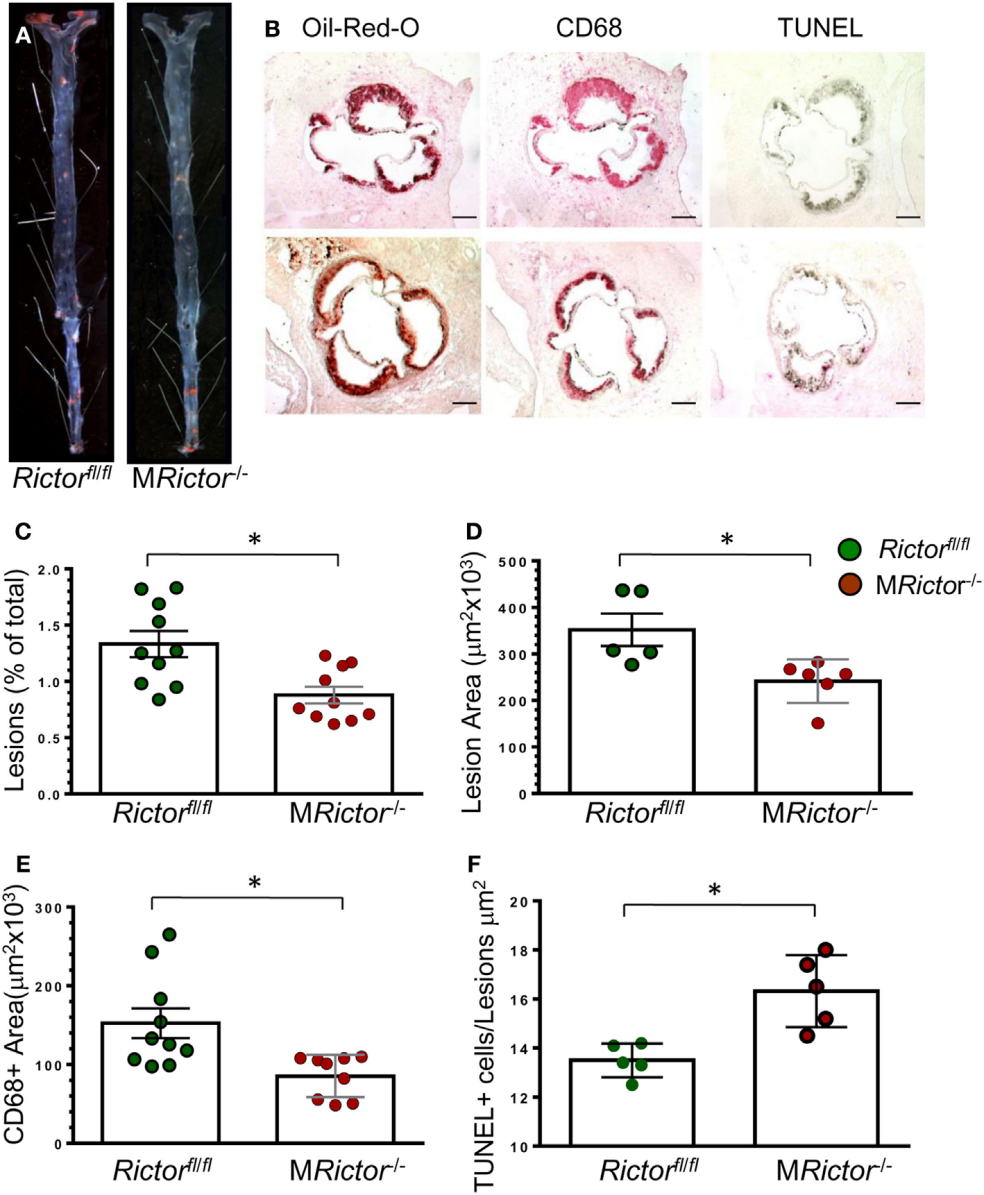
Male M*Rictor*^−/−^ → *Ldlr*^−/−^ mice had less atherosclerosis, smaller macrophage area and more apoptotic cells in the lesion area than control *Rictor*^fl/fl^ → *Ldlr*^−/−^ mice. **(A)** Representative images of and Sudan IV-stained en face preparation of aortas and **(B)** serial cross sections of aortic sinus stained with Oil-Red-O/hematoxylin, CD68 and TUNEL AP from *Rictor*^fl/fl^ → *Ldlr*^−/−^ [**(B)** top panel] and M*Rictor*^−/−^ → *Ldlr*^−/−^ [**(B)** bottom panel] mice. Scale bars, 200 μm. **(C,D)** Quantitation of atherosclerotic lesions in aortas en face and cross sections of aortic sinus of *Rictor*^fl/fl^ → *Ldlr*^−/−^ and M*Rictor*^−/−^ → *Ldlr*^−/−^ mice; *p < 0.05 by Mann–Whitney rank sum test. **(E,F)** Macrophage area stained with MOMA-2 and number of TUNEL + cells in atherosclerotic lesions of mice reconstituted with *Rictor*^fl/fl^ or M*Rictor*^−/−^ bone marrow cells; *p < 0.05 by *t*-test.

In a second experiment, 12-week-old male *Ldlr*^−/−^ mice were lethally irradiated and transplanted with male *Rictor*^fl/fl^ (control group; *n* = 10) or M*Rictor*^−/−^(*n* = 10) bone marrow cells. Again, after 10 weeks on the Western diet, there were no statistically significant differences in BW, blood glucose and serum lipids between these two groups of recipients (Table 1). In contrast, M*Rictor*^−/−^ → *Ldlr*^−/−^ male recipients had 40% smaller atherosclerotic lesions in pinned out aortas en face than control male *Rictor*^fl/fl^ → *Ldlr*^−/−^ mice (Figures 6A–C). Furthermore, M*Rictor*^−/−^ → *Ldlr*^−/−^ mice also had significantly decreased atherosclerosis in the aortic sinus (42%), a smaller CD68 + macrophage area and more TUNEL-positive cells in the atherosclerotic lesions than lesions of control male *Rictor*^fl/fl^ → *Ldlr*^−/−^ recipients (Figures 6D–F). These results demonstrate that loss of mTORC2 in hematopoietic cells of both female and male recipients significantly increases macrophage apoptosis and diminishes atherosclerosis.

## Discussion

The mTOR pathway plays a central role in sensing environmental cues and regulating cell growth, metabolism (8) and immune responses (3, 4). However, the roles of mTORC2 signaling in monocyte/macrophage proliferation and survival, and in atherogenesis remain unclear. Here we generated myeloid lineage-specific *Rictor* deletion in mice to demonstrate for the first time that mTORC2 signaling is crucial for proliferation and survival of monocytes and macrophages. Rictor deficiency prevents mTORC2 assembly and this causes a dramatic reduction of insulin-mediated Akt S^473^ phosphorylation in macrophages. In addition, suppression of Akt signaling was observed in monocytes and neutrophils of M*Rictor*^−/−^ mice *in vivo*. Ablation of mTORC2 signaling inhibited monocyte and macrophage proliferation. Furthermore, *Rictor* deficiency compromised monocyte and macrophage viability, and these dramatic changes in monocyte/macrophage survival were associated with dramatic reductions of atherosclerosis in male and female *Ldlr*^−/−^ mice *in vivo*.

Mammalian target of rapamycin complex 2 regulates cellular metabolism and previous studies have shown that cell-specific *Rictor* ablation significantly suppresses proliferation of endothelial cells (26), helper T-cells (44) and pancreatic β-cells (20). Our results are consistent with these data and indicate that loss of *Rictor* in monocytes and macrophages suppresses their proliferation causing a diminished response to a proliferative factor, PDGF. Surprisingly, Oh and co-workers (45) recently reported that mTORC2 ablation increased proliferation in peritoneal resident macrophages compared to wild-type cells. This discrepancy with our results may be explained by the fact that we analyzed peritoneal macrophages originating predominantly from monocytes, whereas they focused on tissue-resident peritoneal macrophages. The tissue-resident peritoneal macrophages express F4/80 and GATA6 transcription factor, and they presumably originated from the yolk sac (46). In our experiment, only a small fraction (5%) of peritoneal macrophages expressed F4/80 after two days in culture. Moreover, the same group also showed that *Rictor* null monocyte-derived peritoneal macrophages have less proliferation than wild-type cells (45) and this finding is consistent with our results.

Although loss of Rictor in macrophages appears to only modestly affect basal Akt activity, a fact that has been noted previously in several different types of cells (16, 18, 20, 23), the response to insulin was significantly suppressed in M*Rictor*^−/−^ compared to *Rictor*^fl/fl^ macrophages (Figures 1A–C). Moreover, there was an increase in apoptotic monocytes and neutrophils in blood of M*Rictor*^−/−^ mice (Figures 3A,B). More importantly, our *in vitro* experiments convincingly demonstrated that M*Rictor*^−/−^ monocytes and macrophages exhibit compromised viability and generate more apoptosis under conditions of ER stress than *Rictor*^fl/fl^ cells (Figures 3C–F). These data support the idea that mTORC2 with Akt S^473^ phosphorylation are essential for pro-survival signaling (6) and that the failure of this response under conditions of ER stress leads to early apoptosis. Previously we have shown that *IKK*α^−/−^ macrophages exhibited a similar defect in Akt S^473^ phosphorylation and a related increase of apoptosis (30). It is known that IKKα interacts with Rictor and that chemical inhibition or genetic suppression of IKK significantly diminishes mTORC2 activity and Akt signaling (47). Low levels of white blood cells and reduced proliferation of macrophages have been associated with reduced atherosclerosis. Interestingly, proliferation of macrophages in the artery wall has been reported to be an important determinant of atherogenesis (48). Our current findings establish that M*Rictor*^−/−^ mice exhibited relatively low levels of white blood cells, and this is due, at least in part, to decreased proliferation and compromised viability of both monocytes and neutrophils. Furthermore, we cannot exclude the possibility that MRictor^−/−^ neutrophils or monocytes may have an inefficient or delayed response for mobilization from the bone marrow. It remains unclear whether the reduced numbers of T- and B-cells in the blood of M*Rictor*^−/−^ mice are causally related to the reduced numbers or functions of myeloid cells.

Macrophages may be polarized into two distinct subtypes including classically activated M1 and alternatively stimulated M2 macrophages (49, 50). A recent concept of the immune response proposes that Akt-mTORC1 signaling controls polarization by changing metabolism of macrophages (5, 51, 52). For example, LPS treatment induces a switch to high aerobic glycolysis, fatty acid synthesis and a truncated citric acid cycle (the Krebs cycle) to form the M1 pro-inflammatory phenotype. In contrast, IL-4 induces oxidative phosphorylation to generate M2 macrophages for tissue remodeling, immunosuppression and phagocytosis. Both of these shifts generate many metabolites that are acting as signaling molecules with consequent alteration of the downstream immune response. In this context, mTORC1 is considered as a major mediator of the cellular response to stress (53). Loss of tuberous sclerosis 1 leads to constitutive mTORC1 activation in macrophages making them resistant to M2 polarization and producing an increased inflammatory response (54). Accordingly, *Raptor* deficiency in macrophages reduced chemokine gene expression (55) and loss of mTORC1 in hematopoietic cells impaired myelopoiesis at steady state with dampening of some innate immune responses (56). Thus, Akt-mTORC1 signaling is coupled to metabolic input to regulate a key enzyme of Acetyl-CoA synthesis, leading to increased histone acetylation and induction of a subset of M2 genes in macrophages (57).

Compared to mTORC1, our understanding of the role of mTORC2 in macrophage polarization is less well defined. Brown and co-workers (58) were the first to demonstrate that mTORC2 negatively regulates the Toll-like receptor 4-mediated inflammatory response acting via Forkhead box protein O1, a transcription factor regulating glucose metabolism. Festuccia and co-authors later reported that *Rictor* deletion induces M1 macrophage polarization potentiating pro-inflammatory responses (41). More recent studies revealed that loss of *Rictor* inhibits the generation of M2 macrophages while preserving the production of classically activated M1 cells (42). Furthermore, mTORC2 operates in parallel with the STAT6 pathway to facilitate increased glycolysis during M2 activation (43).

Depending on the degree of activation, PI3K-Akt signaling crucially contributes to macrophage polarization with subsequent activation or dampening the immune response (59). The individual Akt isoforms promote distinct polarization phenotypes in macrophages. For example, macrophage *Akt1* ablation induces M1, whereas *Akt2* deficiency promotes the M2 phenotype (60). Recently we have shown that *Akt2*^−/−^ macrophages express significantly lower levels of inflammatory genes and display the M2 phenotype with a suppressed ability for M1 polarization and CCR2 induction (61). In addition to the ratio of Akt1/Akt2 isoforms, the levels of Akt signaling are also crucial for macrophage polarization. For example, overexpression of the PI3K-Akt pathway caused by myeloid cell-specific deficiency of SH2-containing inositol phosphatase or phosphatase and tensin homolog (PTEN), which are negative regulators of PI3K signaling, significantly promote M2 macrophage differentiation decreasing inflammatory cytokine production in macrophages (62–64). In contrast, genetic deficiency of *IKK*α as well as pharmacologic inhibition of IKK, significantly reduces Akt S^473^ phosphorylation via suppression of mTORC2 signaling, and this is accompanied by an M1-related increase in the expression of inflammatory genes (30). Our current results are consistent with these data and indicate that suppression of Akt signaling increases of inflammatory gene expression in M*Rictor*^−/−^ macrophages. Moreover, further suppression of signaling with a low dose of Akt inhibitor IV additionally increased cytokine gene expression levels. Importantly, the genetic loss of Raptor, the main component of mTORC1, eliminated this increase, indicating that mTORC1 mediates the increased expression of these pro-inflammatory genes. Together these data support the concept that the PI3K-Akt-mTOR pathway is critical for restriction of the inflammatory reaction and that overexpression of this signaling pathway reduces macrophage activation by LPS, whereas the inhibition of Akt signaling significantly augments NF-κB activity (65). Furthermore our results suggest an opportunity to pharmacologically control Akt signaling activity and polarization of macrophages in order to modulate innate or adaptive immune responses.

Atherosclerosis remains the number one cause of death and disability in modern societies. Surprisingly, relatively little is known about the role of mTORC2 in atherosclerosis. One can propose mechanisms by which loss of mTORC2 might promote atherosclerosis, including increased inflammatory cytokines in response to LPS or increased production of damage-associated molecular patterns; whereas reduced proliferation and increased apoptosis of macrophages would be predicted to reduce the extent of early atherosclerosis. Therefore, we examined the impact of mTORC2 elimination in hematopoietic myeloid cells on atherosclerosis *in vivo*. M*Rictor*^−/−^ → *Ldlr*^−/−^ recipients had smaller atherosclerotic lesions enriched in apoptotic cells compared to *Rictor*^fl/fl^ → *Ldlr*^−/−^ mice. Thus, loss of *Rictor* in hematopoietic myeloid cells significantly suppressed atherosclerosis in *Ldlr*^−/−^ mice, and these results demonstrate for the first time that macrophage mTORC2 plays a critical role of in atherogenesis. The reduction of atherosclerotic lesions was likely mediated through several inter-related mechanisms such as the inhibition of proliferation and suppression of viability of monocytes that cause monocytopenia in M*Rictor*^−/−^ → *Ldlr*^−/−^ mice. In addition, we found decreased proliferation and viability of M*Rictor*^−/−^ macrophages, which are the major type of cells atherosclerotic lesions. These results are consistent with the current concepts that low numbers of circulating monocytes and high sensitivity of macrophages to pro-apoptotic stimuli are key drivers that suppress atherogenesis (66, 67). Our study also suggests a potential role of targeted inhibition of mTORC2 in monocytes and macrophages as a novel approach for the treatment of atherosclerotic cardiovascular disease.

## Author Contributions

VB. designed and performed experiments, analyzed data, and wrote the manuscript; JH, LD, and YZ performed experiments and analyzed data; JM critically revised the manuscript; and ML supervised the project, designed the studies, analyzed data, and revised the manuscript.

## Conflict of Interest Statement

The authors declare that our research was conducted and performed in the absence of any commercial or financial relationships that could be construed as a potential conflict of interest.
